# Association of the mtDNA m.4171C>A/*MT-ND1* mutation with both optic neuropathy and bilateral brainstem lesions

**DOI:** 10.1186/1471-2377-14-116

**Published:** 2014-05-28

**Authors:** Chiara La Morgia, Leonardo Caporali, Francesca Gandini, Anna Olivieri, Francesco Toni, Stefania Nassetti, Daniela Brunetto, Carlotta Stipa, Cristina Scaduto, Antonia Parmeggiani, Caterina Tonon, Raffaele Lodi, Antonio Torroni, Valerio Carelli

**Affiliations:** 1 UOC Clinica Neurologica, IRCCS Istituto delle Scienze Neurologiche di Bologna, Ospedale Bellaria, Bologna, Italy; 2 Unità di Neurologia, Dipartimento di Scienze Biomediche e NeuroMotorie (DIBINEM), Università di Bologna, Bologna, Italy; 3 Dipartimento di Biologia e Biotecnologie “L. Spallanzani”, Università di Pavia, Pavia, Italy; 4 UOC Neuroradiologia, IRCCS Istituto delle Scienze Neurologiche di Bologna, Ospedale Bellaria, Bologna, Italy; 5 Unità di RMN Funzionale, Policlinico S.Orsola-Malpighi, Dipartimento di Scienze Biomediche e NeuroMotorie (DIBINEM), Bologna, Italy

**Keywords:** Mitochondrial disease, Vision loss, Bilateral brainstem lesions, LHON, mtDNA mutation, Leigh syndrome, Idebenone

## Abstract

**Background:**

An increasing number of mitochondrial DNA (mtDNA) mutations, mainly in complex I genes, have been associated with variably overlapping phenotypes of Leber’s hereditary optic neuropathy (LHON), mitochondrial encephalomyopathy with stroke-like episodes (MELAS) and Leigh syndrome (LS). We here describe the first case in which the m.4171C>A/*MT-ND1* mutation, previously reported only in association with LHON, leads also to a Leigh-like phenotype.

**Case presentation:**

A 16-year-old male suffered subacute visual loss and recurrent vomiting and vertigo associated with bilateral brainstem lesions affecting the vestibular nuclei. His mother and one sister also presented subacute visual loss compatible with LHON. Sequencing of the entire mtDNA revealed the homoplasmic m.4171C>A/*MT-ND1* mutation, previously associated with pure LHON, on a haplogroup H background. Three additional non-synonymous homoplasmic transitions affecting ND2 (m.4705T>C/*MT-ND2* and m.5263C>T/*MT-ND2*) and ND6 (m.14180T>C/*MT-ND6*) subunits, well recognized as polymorphisms in other mtDNA haplogroups but never found on the haplogroup H background, were also present.

**Conclusion:**

This case widens the phenotypic expression of the rare m.4171C>A/*MT-ND1* LHON mutation, which may also lead to Leigh-like brainstem lesions, and indicates that the co-occurrence of other ND non-synonymous variants, found outside of their usual mtDNA backgrounds, may have increased the pathogenic potential of the primary LHON mutation.

## Background

Leber’s hereditary optic neuropathy (LHON), a maternally inherited blinding disorder, is associated with three common mitochondrial DNA (mtDNA) point mutations affecting complex I (m.3460G>A/*MT-ND1*, m.11778G>A/*MT-ND4*, m.14484T>C/*MT-ND6*) in 90% of cases [1]. A wider range of mtDNA mutations, again mainly in complex I genes (*MT-ND1*, *MT-ND5* and *MT-ND6;*http://www.mitomap.org/), have been instead associated with variably overlapping phenotypes of LHON, mitochondrial encephalomyopathy with stroke-like episodes (MELAS) and Leigh syndrome (LS) [2]. Occasionally, the latter manifestations have been described also with the common LHON mutations [3]. A recent study, reviewing the neuroradiological features of a large cohort of LS patients, suggested that bilateral brainstem lesions are more typical of mtDNA-related complex I deficiency, in most cases with a concurrent striatal involvement [4]. We have recently reported one such case with brainstem lesions without striatal involvement associated with the rare m.3890G>A/*MT-ND1* mutation [5].

We here describe for the first time a maternal lineage carrying the homoplasmic rare m.4171C>A/*MT-ND1* mutation, previously associated only with pure LHON, whose proband in addition to optic neuropathy presented acute onset of vomiting, vertigo and nystagmus in association with bilateral brainstem lesions affecting vestibular nuclei.

## Case presentation

**Figure 1:**
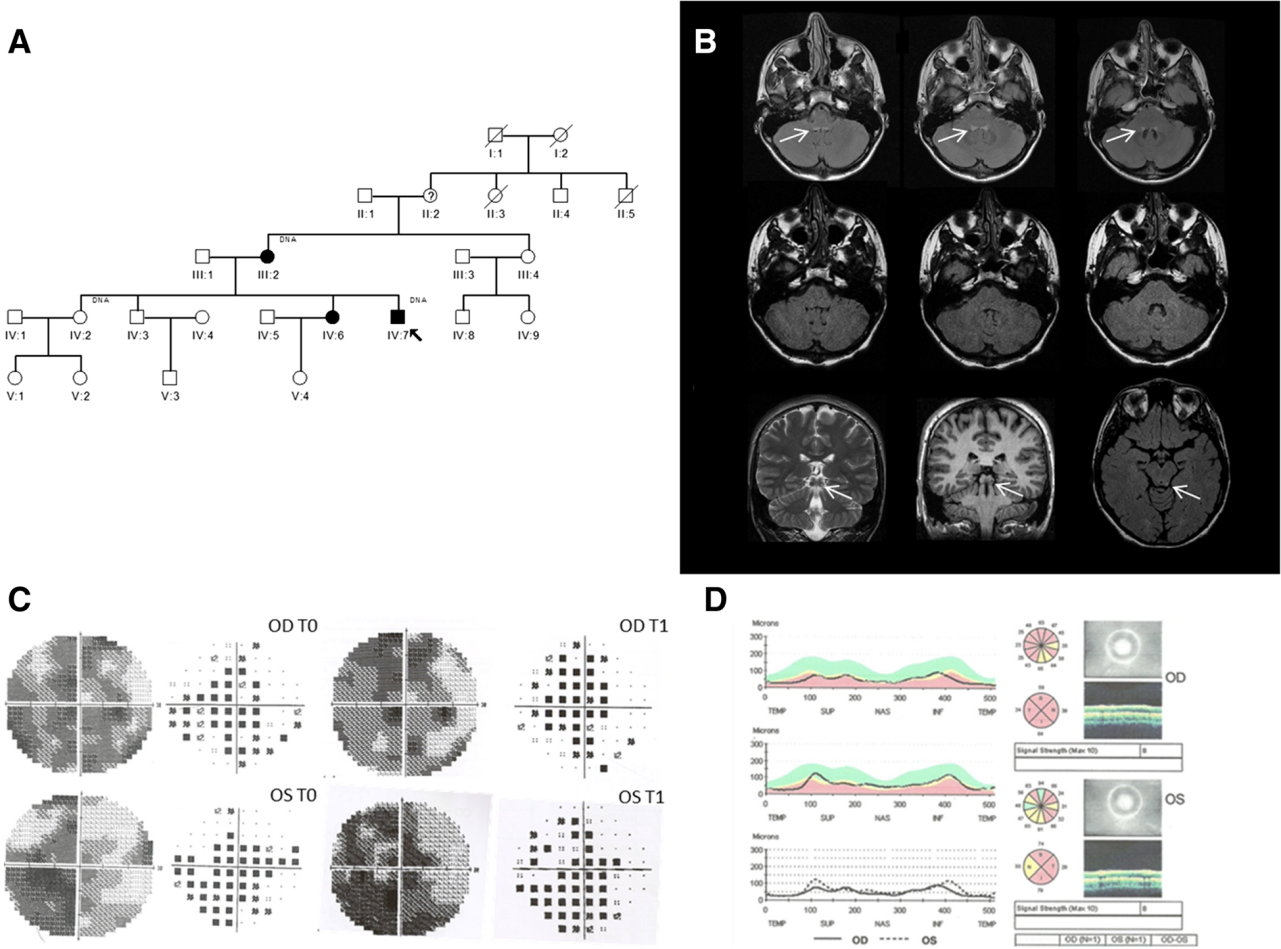
**Pedigree, brain MRI and ophthalmologic findings. A**: pedigree with affected individuals (blackened symbols) and individuals for which DNA analysis was performed (DNA). **B**: Axial FLAIR T2-weighted brain MRI at baseline showing bilateral vestibular nuclei lesions (upper row, arrows), their disappearance at follow-up (middle row) in association with the appearance of a lesion involving the left inferior colliculus (lower row, arrow). **C**: Humphrey visual fields (total deviation) of both eyes at baseline (T0) and follow-up (T1). A slight increase in mean deviation (MD) from T0 to T1 is reflected by shrinkage of the scotoma, with some improvement of the peripheral field sensitivity. **D**: optical coherence tomography showing diffuse optic atrophy in both eyes, with some preservation of the nasal quadrant in OS.

A 16-year-old male of Belarusian origin was referred for the acute onset of vomiting and vertigo. Family history was relevant for the recurrence of subacute visual loss and optic atrophy in the proband’s mother (at age 17) and one sister (at age 9) (Figure 1A), both reporting a spontaneous visual recovery.

The proband suffered subacute loss of vision bilaterally at age 12, after a febrile illness, without recovery. We observed this patient at age 16, three months after the acute onset of recurrent vomiting and vertigo. Neurological examination at admission showed horizontal nystagmus, segmental and parcellar myoclonic jerks at upper and lower limbs, slight dysmetria at the right upper limb, weak deep tendon reflexes, positive Romberg sign and mild ataxic gait.Brain magnetic resonance imaging (MRI) revealed bilateral lesions of the brainstem involving the vestibular nuclei (Figure 1B, upper row). Moreover, MRI showed atrophy of the optic nerves bilaterally with hyperintense signal in the T2-weighted images with enhancement after gadolinium administration (not shown). Serum lactic acid after standardized exercise was abnormally elevated (3.1 mM, n.v. 1–2 mM). Ophthalmologic evaluation showed bilateral temporal pallor, visual acuity (a measure of central vision function) was 0.2 bilaterally and visual fields demonstrated bilateral cecocentral scotomas, more severe in OD (Figure 1C). Optical coherence tomography evaluating retinal nerve fiber layer thickness showed bilateral diffuse optic atrophy with nasal sparing (Figure 1D). Cognitive functions assessment was normal, as well as audiometry and electroencephalography. A therapy with high dose idebenone (540 mg/day) was started with improvement of vomiting and vertigo and visual function after about two months.Follow-up evaluation after eight months from therapy start showed an improvement of visual acuity in OS (0.4) and a slight improvement of visual field defect in both eyes (mean deviation from −18 to −15 in OD and −16 to −15 in OS) (Figure 1C). Lactic acid after exercise was slightly worse compared to the baseline evaluation (3.9 mM), but the patient reported intense physical training in the previous month. Brain MRI follow-up showed a complete resolution of vestibular nuclei lesions (Figure 1B, middle row), and the appearance of a new lesion affecting the left tuberculus quadrigeminus (Figure 1B, lower row).

We first surveyed the proband for the three common LHON mutations, which were absent, then his entire mtDNA was sequenced (GenBank accession number KF927040). The mitogenome was characterized by the diagnostic mutational motif of haplogroup H (http://www.phylotree.org/). In addition, it also harbored the m.4171C>A/*MT-ND1* transversion relative to the revised Cambridge Reference Sequence, [6] which causes the p.L289M amino acid change and was previously reported in association only with a “pure” LHON phenotype, and four transitions: the synonymous m.3798C>T/*MT-ND1* and the three non-synonymous changes m.4705T>C/*MT-ND2*, m.5263C>T/*MT-ND2* and m.14180T>C/*MT-ND6*, all affecting complex I. Restriction fragment length polymorphism (RFLP) analysis of the proband, one unaffected sister and the mother (Figure 1A) on DNA extracted from both blood cells and urinary epithelium sediment confirmed that the m.4171C>A/*MT-ND1* transversion was consistently homoplasmic. As for the three non-synonymous changes, they cause the amino acid substitutions p.M79T and p.A265V in ND2, and p.Y165C in ND6, but they all have been reported as diagnostic markers for some ancient continent-specific haplogroups or sub-haplogroups: m.4705T>C/*MT-ND2* for sub-haplogroup F1b1a1a1, m.5263C>T/*MT-ND2* for sub-haplogroups V1a1, U5a1d1, F4, B4a1c3a, M29a, M2b1b, G1a1a3, L2a5, and m.14180T>C/*MT-ND6* for sub-haplogroups T2b26, D4b1a1a, L2a1l2a (http://www.phylotree.org/). Finally, all three amino acid residues are moderately conserved [7] and *in silico* analysis predicted a possibly pathogenic role for the m.14180T>C/*MT-ND6* and m.4705T>C/*MT-ND2* variants (Table 1). RFLP survey of these three mutations confirmed that they were homoplasmic in the mother and one unaffected sister. We also extended the conservation analysis on the protein regions surrounding these polymorphisms, according to a previously established method [8]. All three changes had an invariant amino acid within four contiguous amino acidic positions (+4/−4 aa) and they hit a protein region with a local conservation higher than global conservation of the protein subunit (Table 1). Finally, we compared the mtDNA background of our proband with those of the previous cases reported with the m.4171C>A/*MT-ND1* transversion (Table 2) [8-11]. Only one pedigree from China, characterized by almost complete penetrance, displayed the co-occurrence of possibly synergistic complex I variants [11].

**Table 1:** Conservation analysis of private variants and contiguous amino acid residues

**Mutation**	**m.4705T>C**	**m.5263C>T**	**m.14180T>C**
**Locus**	*MT-ND2*	*MT-ND2*	*MT-ND6*
**AA change**	p.M79T	p.A265V	p.Y165C
**Conservation (%)**			
**Eukaryotes**	25 (n = 161)	48 (n = 161)	36 (n = 124)
**Vertebrates**	3 (n = 126)	57 (n = 126)	54 (n = 83)
**Mammals**	41 (n = 95)	75 (n = 95)	26 (n = 43)
**Prediction**			
**Polyphen 2**	Benign	Benign	Probably damaging
**Provean**	Deleterious	Neutral	Deleterious
**Contiguous amino acid conservation**			
**Interval (−10/+10)**	69-89	255-275	155-174
**Local conservation (%)**	68	83	69
**Global conservation (%)**	62	62	52
**Nearest invariant position (−/+)**	−1/+2	−1/+1	−1/+4

**Table 2:** Non-synonymous nucleotide changes reported in published mtDNAs with m.4171C>A

**Case**	**Origin**	**Haplogroup**	**Non-synonymous nucleotide changes**^**a**^	**Reference**
1	Korea	A	**4171A**, 4824, 8794, 14766	[9]
2	Korea	A	**4171A**	[9]
3	China	n.a.	n.a.	[10]
4	China	N9a1	**4171A**, *10203*^b,^,12358, *14564*^b^, 14766, *14841*^*c*^, 15095	[11]
5	France	J2b1	**4171A**, 4216, *7632*^c^, 10398, 13708, 14766, 15257, 15452A, 15812	[8]
6	Belarus	H	**4171A**, *4705*^b^, *5263*^b^, *14180*^b^	Present study

^a^The non-synonymous nucleotide changes at nps 8860 (8860G) and 15326 (15326G), present in all sequenced samples, are not included because they are private mutations of the reference sequence.^7^ In *Italics*, variants outside of their usual mtDNA background: ^b)^variants in Complex I subunits or ^c)^variants outside in Complex I subunits.

## Discussion and conclusions

We report the association of the m.4171C>A/*MT-ND1* LHON mutation with bilateral lesions of the brainstem resembling Leigh syndrome associated with acute onset of vomiting and vertigo, in addition to subacute bilateral loss of central vision, typical of LHON. This case extends the clinical features associated with this rare mutation, previously reported only in association with pure LHON, [8-11] and reissues the unexplained wide variability in severity of homoplasmic mtDNA mutations [2]. This family is the sixth maternal lineage carrying this rare mutation, the second on a European mtDNA background. Non-synonymous mtDNA variants, in particular those affecting complex I and III subunit genes, have been implicated in both variable penetrance and severity of LHON clinical expression [7,12]. For example, one LHON family from China carrying the m.4171C>A/*MT-ND1* and the m.14841A > G/*MT-ND6* variant, showed an almost complete penetrance, unusual for LHON [11]. Our genetic analysis also supports the possible role of supplementary mtDNA variants in increasing disease severity leading to the overlapping phenotype of LHON and LS. In fact, our patient’s mitogenome harbored, in addition to the m.4171C>A/*MT-ND1* pathogenic mutation, three non-synonymous variants in ND subunits of complex I. These polymorphic variants are reported in a large variety of sub-haplogroups, but never within the context of haplogroup H, as in our patient. Increasing evidence shows that some non-synonymous variants may be tolerated in certain haplogroups and exert a different functional effect in others. For example, the non-synonymous variant m.3394T>C/*MT-ND1* (Y30H) has been associated with LHON in the Asian B4c and F1 haplogroup backgrounds, whereas it is enriched as a common variant on the M9 background in Tibet and the C4a4 background on the Indian Deccan Plateau, exerting an adaptive role to high-altitude in Tibetan and Indian populations, respectively [13]. It is increasingly recognized that genetic variation characterizing mtDNA haplogroups exerts slight but significant functional differences, as demonstrated by studies on cybrid cell lines carrying mtDNA from control subjects with different haplogroups [14]. Thus, the peculiar coexistence of the three variants with the m.4171C>A/*MT-ND1* mutation may explain the occurrence of the severe phenotype combining LHON and brainstem lesions.

Furthermore, our proband is a male, whereas the other two affected individuals in the same maternal lineage are females and they both had a disease limited to the optic nerve with spontaneous partial regression. This is compatible with the higher predisposition of males to LHON and with the protective role of oestrogens in female mutation carriers, as we recently demonstrated [15].

Idebenone administration coincided with a complete resolution of the brainstem lesions and clinical symptoms, with also a slight improvement in visual function. However, this observation must be evaluated with caution given the possibility of spontaneous visual recovery in LHON [1]. The patients from the previously reported Korean and French families with the m.4171C>A/*MT-ND1* were also characterized by a high rate of spontaneous recovery of visual acuity [8,9]. Furthermore, we previously observed another case resembling our proband who also presented spontaneous regression of the vestibular nuclei lesions before idebenone administration [5]. Interestingly, this patient had another rare mutation (m.3890G>A/*MT-ND1*) affecting complex I, associated with both LHON and Leigh phenotypes and presented in association with the regression of the vestibular nuclei abnormalities an involvement of the inferior colliculi, identical to the unilateral involvement the left inferior colliculus showed in our patient (Figure 1) [5].

In conclusion, homoplasmic mtDNA missense mutations affecting ND subunits of complex I display a wide variability in penetrance and clinical severity. The genetic variation of mtDNA may contribute more substantially than previously recognized to this variability, raising the issue whether complete mtDNA sequencing should be carried out routinely in these patients. Further nuclear DNA variation and complex interactions with environmental factors are probably also relevant, but these are currently poorly understood modulators.

## Consent

Written informed consent was obtained from the patient for publication of this Case report and any accompanying images. A copy of the written consent is available for review by the Editor of this journal.

## Abbreviations

LHON: Leber’s hereditary optic neuropathy; LS: Leigh syndrome; MELAS: Mitochondrial encephalomyopathy, lactic acidosis, and stroke-like episodes; MRI: Magnetic resonance imaging; mtDNA: Mitochondrial DNA; OD: *oculus destrum*; OS: *oculus sinistrum*; RFLP: Restriction fragment length polymorphism.

## Competing interests

All the authors declare that they have no competing interests.

## Authors’ contributions

CLM carried out the clinical evaluation, participated to the study design, drafted and revised the manuscript. LC carried out the molecular genetic studies, participated to the study design, drafted and revised the manuscript. FG carried out the mtDNA genetic analysis. AO performed the sequence alignment. FT performed MRI analysis. SN, DB, CS, CS and AP contributed to the clinical evaluation and management. CT carried out MRI analysis. RL participated in the study design and revised the manuscript. AT participated in the study design, drafted and revised the manuscript. VC designed and supervised the study, drafted and revised the manuscript. All authors read and approved the final manuscript.

## Pre-publication history

The pre-publication history for this paper can be accessed here:

http://www.biomedcentral.com/1471-2377/14/116/prepub
